# Reducing Obesogenic Eating Behaviors in Hispanic Children through a Family-Based, Culturally-Tailored RCT: *Abriendo Caminos*

**DOI:** 10.3390/ijerph19041917

**Published:** 2022-02-09

**Authors:** Maribel Barragan, Viridiana Luna, Amber J. Hammons, Norma Olvera, Kimberly Greder, Flavia Cristina Drumond Andrade, Barbara Fiese, Angela Wiley, Margarita Teran-Garcia, the Abriendo Caminos Research Team

**Affiliations:** 1 Division of Nutritional Sciences, University of Illinois at Urbana-Champaign, Urbana, IL 61801, USA; maribel4@illinois.edu (M.B.); viridiana.luna@actforchildren.org (V.L.); 2 Department of Child and Family Science, California State University, Fresno, CA 93740, USA; ahammons@csufresno.edu; 3 Department of Psychological, Health, and Learning Sciences, University of Houston, Houston, TX 77204, USA; Nolvera@Central.UH.EDU; 4 Department of Human Development and Family Studies, Iowa State University, Ames, IA 50011, USA; kgreder@iastate.edu; 5 School of Social Work, University of Illinois at Urbana-Champaign, Urbana, IL 61801, USA; fandrade@illinois.edu; 6 Department of Human Development and Family Studies, Family Resiliency Center, University of Illinois at Urbana-Champaign, Urbana, IL 61801, USA; bhfiese@illinois.edu; 7 Department of Human Development and Family Science, Auburn University, Auburn, AL 36849, USA; awiley1@auburn.edu; 8 Integrated Health Disparities Programs, University of Illinois Extension, Champaign, IL 61820, USA; 9 Carle Illinois College of Medicine, Family Resiliency Center, University of Illinois at Urbana-Champaign, Urbana, IL 61801, USA

**Keywords:** childhood obesity, Hispanic families, behavior change, family-based intervention, nutrition education, culturally-tailored programs

## Abstract

Family-based interventions that incorporate culturally-tailored multi-component curricula and are grounded on evidence-based information and theoretical frameworks can help reduce the prevalence of obesity among Hispanic children. *Abriendo Caminos*: Clearing the Path to Hispanic Health is a multi-site culturally-tailored randomized control trial that aims to reduce obesity rates in Hispanic families by delivering education on nutrition, family wellness, and physical activity. This study evaluated the effect of the *Abriendo Caminos* six-week intervention on dietary behaviors of Hispanic children (6–18 years). Mothers (*n* = 365) reported their child’s eating behavior intake using the U.S. Department of Education’s Early Childhood Longitudinal Study protocol (ECLS). Pre/post dietary changes were evaluated using separate generalized estimating equation models adjusted for site, child sex, and child age group. Findings indicate a reduction in the frequency of sugar-sweetened beverages (OR 0.55, 95% CI 0.35, 0.87, *p* = 0.01), French fries (OR 0.56, 95% CI 0.36, 0.86, *p* = 0.009), and fast food (OR 0.55, 95% CI 0.36, 0.84, *p* = 0.006) consumption among children in the intervention arm. Additionally, children in the intervention arm increased their frequency of vegetable consumption (OR 1.84, 95% CI 1.08, 3.12, *p* = 0.03). The *Abriendo Caminos* intervention effectively improved four of eight eating behaviors in a short-term intervention.

## 1. Introduction

A range of studies shows that Hispanic children in the U.S. exhibit disproportionately high rates of obesity compared to children of other racial/ethnic groups [1,2,3,4]. Approximately 26% of Hispanic, 22% of non-Hispanic Black, and 14% of non-Hispanic White children are impacted by pediatric obesity [1]. In turn, pediatric obesity can lead to various physical and psychological medical consequences that often persist into adulthood [5]. These include the development of chronic metabolic diseases, eating disorders, and depression [6,7,8]. Identifying effective strategies to prevent childhood obesity has been challenging due to its complex nature. The causes of obesity are multi-faceted and include the interactions between eating behaviors (e.g., frequency, amount, and eating occasions), dietary intake (e.g., dietary patterns and food components), physical inactivity, obesogenic environments, and genetics [5,7]. Family-based lifestyle interventions that target multiple modifiable lifestyle behaviors show promise in preventing obesity through the adoption of healthy behaviors [9,10]. However, more evidence-based interventions that target the various etiologies of obesity incorporate cultural values and involve the family unit are warranted for the Hispanic community.

Modifiable lifestyle factors such as unhealthy eating behaviors, dietary patterns, and physical inactivity can play a critical role in the development of obesity and its associated metabolic diseases. Eating behaviors, such as the frequent consumption of refined grains, processed foods, fast food, candy, and sugar-sweetened beverages (SSBs), have been associated with the development of childhood obesity [11]. Conversely, dietary patterns rich in fruits, vegetables, legumes, lean proteins, nuts, unsaturated fats, and whole grains have been associated with a decreased risk of obesity and all-cause mortality [12]. Epidemiological studies have found that Hispanic children have better dietary patterns than children from other ethnic groups [13,14,15]. However, SSB consumption is prevalent, and this group has the highest sodium consumption and intake of added sugar [13,14,16]. Moreover, less than half of Hispanic youth meet the daily recommendations for vegetables, legumes, or whole-grain intake [13]. Cross-sectional studies have also found that Hispanic children are not meeting physical activity recommendations [17,18]. Physical inactivity among the Hispanic community is a result of multiple factors, including lack of time, emotional and physical exhaustion due to long hours in physically demanding jobs, lack of accessible spaces, and unsafe neighborhoods [19,20]. The improvement of eating behaviors and participation in physical activity have become core components for interventions that aim to reduce the burden of obesity [9,21,22].

Key aspects of successful obesity prevention interventions for the Hispanic community include culturally-tailored curricula, a theoretical framework, acknowledgment of structural and social determinants of health, and multiple modifiable lifestyle factors [9,10,23,24]. Culturally-tailored interventions that are available in Spanish, incorporate cultural values, and include culturally appropriate foods have demonstrated strong engagement among Hispanic families [9]. Additionally, the inclusion of the family unit can result in more successful outcomes as the Hispanic culture values strong family ties [9].

The *Abriendo Caminos: Clearing the Path to Hispanic Health* (hereafter referred to as *Abriendo Caminos,* which means “opening roads”) parallel-arm, randomized controlled intervention is an evidence-based, primary obesity prevention program that delivers education on nutrition, family wellness, and the benefits of physical activity [25]. *Abriendo Caminos* is designed for Hispanic families and is grounded in the applied behavior theory for community nutrition programs and tenets of the social cognitive theory [26,27,28]. *Abriendo Caminos* focuses on targeting multiple areas of wellbeing (nutrition, family wellness, physical activity). Each nutrition lesson delivers education on a major food group and includes interactive activities that incorporate regionally adapted cultural elements of a Hispanic diet. Other details of the intervention have been published [25]. Preliminary results indicate improved dietary patterns of mothers who participated in the *Abriendo* *Caminos* intervention [29].

This study aims to evaluate the effect of *Abriendo Caminos* on the outcome of child-eating behaviors by measuring changes in the frequency of consumption from baseline to post-intervention. It is hypothesized that the eating behaviors (frequency of consumption) of nutrient-dense foods such as fruits and vegetables will increase, and consumption of energy-dense food/beverages such as fast food and SSBs will decrease among children in the intervention arm compared to those in the control group at six weeks post-intervention.

## 2. Materials and Methods

### 2.1. Study Design

*Abriendo Caminos* is a multi-site, longitudinal, randomized controlled intervention designed for Mexican and Puerto Rican families that aims to prevent obesity (Clinicaltrials.gov Identifier: NCT03505658). Trained health, nutrition, and food science, child and family development, and kinesiology students, Cooperative Extension educators, community partners, and research staff delivered the intervention at five sites: Illinois, California, Iowa, Texas, and Puerto Rico. The present study reports on data from four of the five sites: Illinois (University of Illinois at Urbana-Champaign), California (California State University, Fresno), Texas (University of Houston), and Iowa (Iowa State University). Data from Puerto Rico were not included in this study because hurricanes Maria and Irma and the COVID-19 pandemic delayed data collection. Parents and children signed written informed consent and assent, respectively, prior to participating in any study data collection protocols. This study was conducted according to the guidelines of the Declaration of Helsinki and approved by the Institutional Review Board of the University of Illinois at Urbana Champaign (#15503, 4 October 2015). Annual renewals are conducted. The institutional review boards at the participating academic institutions approved study protocols.

### 2.2. Participants

Between the fall of 2015 and spring of 2019, Hispanic families were recruited by word of mouth and/or flyers at churches, schools, flea markets, and through Hispanic serving organizations. There was a pre-planned, staggered site project initiation over that time. 

Eligible participants included Mexican and Puerto Rican families (at least one parent had to self-identify as Mexican or Puerto Rican) with a child or adolescent between the ages of 6 and 18 years. Families were randomized into either the intervention or the control arm on the first day of data collection. Participants were randomized using a random number generator (Research Randomizer, Lancaster, PA, USA, 2013) or by pulling a ball from a bag, where the color corresponded to a specific treatment arm. The two randomization methods were implemented consistently per site and workshop cycle, in response to community feedback. 

### 2.3. Intervention

The *Abriendo Caminos* intervention used a family-based approach to deliver culturally-tailored nutrition education, family wellness, and physical activity workshops. A detailed description of how the program was developed and its objectives have been published elsewhere [25,29]. Families randomized to the intervention arm participated in six weekly workshops. Each workshop session lasted two hours and included three components: nutrition (30 min), family wellness (30 min), and physical activity (60 min). Because the focus of this report is on eating behavior outcomes, only the nutrition curriculum is described in detail. The curriculum was developed based on the 2015–2020 Dietary Guidelines for Americans and MyPlate recommendations [30,31]. Mothers and children attended separate nutrition classes that covered the same topic simultaneously. The mother’s nutrition class was delivered in Spanish, while the children’s nutrition class was delivered in English. The weekly topics covered are presented in Table 1. 

The *Abriendo Caminos* curriculum targeted each of the five food groups, barriers to making healthier dietary choices, and methods to make realistic dietary changes. The children’s nutrition classes were tailored to different literacy levels and incorporated hands-on activities as well as culturally relevant food demonstrations. For example, during the fruit and vegetable lesson, guava, mangos, beets, rutabaga, or jicama were provided to the children, and they learned about the importance of each in Hispanic cuisine. A food demonstration accompanied each nutrition lesson to reinforce nutrition knowledge and expose children to new foods and new ways to prepare familiar foods. Children were exposed to a variety of fruits and vegetables in many different forms. For example, jicama was presented plain, with lime juice, or chili powder seasoning. Children were able to try all varieties and select which was their favorite. A key concept throughout the nutrition class was *más o menos* (a little more and a little less) [25]. This concept focused on consuming more nutrient-dense foods and less energy-dense foods. Nutrition lessons were complemented by providing educational material/handouts in Spanish and English. 

The last workshop (week 6), Fiesta, was a celebration and graduation for the families. The families were encouraged to use the information they learned in the workshops to modify a traditional family recipe and share it with the other participants. Families employed modifications such as baking instead of frying, substituting lard with healthy oils, and drinking infused water instead of beverages with added sugars. Families in the intervention arm were encouraged to invite all family members to the workshop intervention, but data were only collected from one parent and one target child.

### 2.4. Control

Families randomized to the control arm completed T0 and T1, baseline and six weeks post-intervention, questionnaires but did not participate in the weekly workshops. At T0, control families were notified that educational materials would be disseminated after completing T1 protocols. The educational material consisted of a DVD of culturally appropriate physical activity and educational handouts similar to those used in the weekly workshops. 

Participants from both arms received incentives for completing baseline (T0, $25) and six weeks post-intervention (T1, $30) measures. Families in the intervention arm received an additional incentive ($10) for each workshop they attended, and a bonus of $20 if they attended all six workshops. 

### 2.5. Measures

#### 2.5.1. Child Dietary Intake

The main outcome was changes in children’s eating behaviors by measuring the frequency of consumption of various foods as reported by the target parent at T0 and T1. All questionnaires were available in Spanish or English and bilingual trained research assistants were available to assist mothers in completing the questionnaire or answering questions. Mothers completed the 8-item food consumption survey from the U.S. Department of Education’s Early Childhood Longitudinal Study (ECLS) at T0 and T1 [32]. The questionnaire assessed the consumption frequency of eight food behaviors: “1. SSB (non-diet soda, non-100% fruit drinks, or sports drinks), 2. 100% fruit juice, 3. fruits (fresh, frozen, canned, or dried), 4. French fries (or other fried potatoes), 5. vegetables (other than fried potatoes), 6. fast food (meal or snack from a restaurant with no wait service), 7. sweets (candy, ice cream, cookies, cakes, brownies), and 8. salty snacks (potato chips, corn chips, pretzels, and crackers) during the past seven days.” Response options included 1 = “once a day,” 2 = “twice a day,” 3 = “three times a day,” 4 = “four or more times a day,” 5 = “1–3 times during the past 7 days,” 6 = “4–6 times during the past 7 days,” 7 = “my child didn’t eat any during the past 7 days,” and 8 = “don’t know.” In order to create a food intake score, responses 5 and 6 were converted to average times per day by dividing the mean by 7 days per week [33]. Response 5 was converted to 0.2857 times per day (2/7 days), and response 6 was converted to 0.7143 times per day (5/7 days) [33]. Response 8 (don’t know) was treated as missing data [33]. The eight eating behaviors were then dichotomized, according to the recommendations of the U.S. Department of Agriculture Dietary Guidelines for Americans, which now include recommendations for children based on age categories [34]. While 100% fruit juice recommendations are limited to eight fluid ounces per day, professional organizations recommend substituting 100% fruit juice with whole fruits [34,35]. Given that energy-dense foods such as SSBs, 100% fruit juice, French fries, fast food, sweets, and salty snacks are not essential components of a healthy diet, they were dichotomized as either consumption 0 or >0 times per day. Fruits and vegetables were dichotomized as <2 or ≥2 times per day. Fruits and vegetables are recognized as essential components of a healthy diet, and it is recommended that children consume 2 and 2.5 servings of fruits and vegetables in a 2000 calorie diet, respectively [34,36]. 

#### 2.5.2. Demographics and Anthropometric Measurements

Demographic data collected at T0 from mothers include child’s date of birth, child’s sex, maternal date of birth, maternal place of birth, marital status, education, and household income, Table 2. Anthropometric data, including the height and weight of mother and child, were collected at T0 and T1 using Seca stadiometers and scales, respectively (Seca North America, Chino, CA, USA). Height was measured in a standing position with both feet touching the base of the board and the head in the Frankfort Plane. Weight was measured in light clothing. Anthropometric measurements were taken twice by trained research staff, and the average of the two measurements was documented. Body mass index (BMI) was calculated by kg/m^2^, and BMI percentiles were determined according to the Centers for Disease Control and Prevention (CDC) guidelines for child sex and age [37].

### 2.6. Statistical Analysis

Descriptive statistics were conducted to examine baseline sociodemographic factors by group (intervention or control). Differences in sociodemographic characteristics by group were assessed using Chi-square tests for categorical variables and *t*-tests for continuous variables. Normality was tested using skewness, kurtosis, and Shapiro–Wilk tests.

A repeated-measure generalized estimating equation (GEE) model was used to evaluate maternal reported changes in the frequency of child consumption of eight food groups. The GEE model is an extension of a generalized linear model that can be used to analyze repeated data as it takes into account within-participant correlation [38]. A binomial model with a logit link was utilized, given that response variables were dichotomized. Results are shown in Table 3. The eight foods were examined in separate models to test the effect of group, time, and group x time interaction and were adjusted for site, child sex, and child age group. Group had two levels (intervention, control), with control being the reference. Similarly, time was a two-level variable (baseline, six weeks) with baseline as the reference. The site variable had four levels (Illinois, California, Iowa, and Texas), with Illinois serving as the reference. Child sex was a two-level variable (boy and girl), with the boy being the reference. The child age group variable was created by dichotomizing children’s age at <144 months (age 12 years) and ≥144 months, with <144 months being the reference. A significant group x time interaction indicated that the change from baseline to six weeks varied according to the group assignment. Table S1 describes maternal reports of children’s eating behaviors before and after the workshops.

Two additional analyses were performed. First, a sensitivity analysis was modeled using the baseline median consumption of each eating behavior as the cutoff point. Results are presented in Table 4. Second, we used multinomial models for scale using the original eight food intake scores, as others have reported [9,33,39]. Results are presented in Table S2. In all analyses, we adjusted for site, child sex, and child age group. All analyses were performed using SAS statistical software package (version 9.4, SAS Institute, Inc, Cary, NC, USA). A two-sided *p* < 0.05 was considered statistically significant.

## 3. Results

### 3.1. Participants

The initial sample consisted of 372 mother–child dyads (211 intervention, 161 control). Eight children did not meet the age criteria (seven children were younger than 66 months and one was older than 228 months) and eight dyads with missing data (one child’s sex and seven children’s date of birth were missing), hence 16 dyads were excluded. As a result, the final sample consisted of 356 mother–child dyads. There were 72 mother–child dyads enrolled in Illinois, 122 mother–child dyads enrolled in California, 73 mother–child dyads enrolled in Iowa, and 89 mother–child dyads enrolled in Texas. There were no differences in children’s age, sex, or BMI percentiles by group (intervention or control). Missing data for mothers’ demographics include age (*n* = 8), BMI (*n* = 5), place of birth (*n* = 3), marital status (*n* = 3), education (*n* = 36), and annual household income (*n* = 36). 

### 3.2. General Descriptive Characteristics

As shown in Table 2, children’s mean age ± standard deviation was 10.2 ± 3.1 and 9.9 ± 2.7 years for the control and intervention group, respectively. Children younger than 12 years of age comprised 71.33% of the control arm and 80.10% of the intervention arm.

More than half of the children in the control (54.67%) and intervention (58.25%) group were girls. Over half of the children were affected by overweight (18.54%) or obesity (35.96%). We did not observe differences in mother’s age, mean BMI, BMI categories, birthplace, marital status, education, or annual household income by group. The mother’s mean age ± standard deviation was 39.3 ± 9.1 and 39.4 ± 7.9 for the control and intervention arm, respectively. The majority of mothers were affected by obesity, with an average BMI of 32.9 ± 6.8 for the control, and 32.0 ± 7.3 for the intervention arm. Most mothers were born outside of the U.S. (predominately in Mexico). Approximately 80% of mothers were either married or living with a partner, and approximately half had at least a high school education. Close to 70% of families had an annual income of less than $30,000.

### 3.3. Baseline Child Eating Behaviors

There were no statistically significant differences in the proportion of children’s dietary consumption of target foods during the previous week between children assigned to the intervention or control group. On average, 82% of children had consumed SSBs more than 0 times per day, 33% of the control group and 49% of the intervention. Similarly, 82% of children had consumed 100% fruit juice more than 0 times per day. Most children had consumed sweets (88%) or salty snacks (91%) more than 0 times per day. Less than half of children had consumed fruit (40%) or vegetables (22%) twice per day, with similar proportion of children in the intervention or control groups. Seventy percent of children had consumed French fries more than 0 times per day, with a higher proportion in the intervention (41%) than the control group (29%), and 71% of the children had consumed fast food more than 0 times per day. Supplementary Table S1 describes in detail maternal reports of children’s eating behaviors before and after the workshops.

### 3.4. Pre- and Post-Intervention Child Eating Behavior Changes

The results of the GEE analysis are shown in Table 3. Parameter estimates are presented for the main effects of group, time, and group x time interaction using binomial models. The frequency of consumption for SSBs (OR 0.55, 95% CI 0.35, 0.87, *p* = 0.01), French fries (OR 0.56, 95% CI 0.36, 0.86, *p* = 0.009), and fast food (OR 0.55, 95% CI 0.36, 0.84, *p* = 0.006) decreased significantly with time among children in the intervention group. Additionally, there was a significant increase in the frequency of vegetable consumption from baseline to six weeks for the children in the intervention group (OR 1.84, 95% CI 1.08, 3.12, *p* = 0.03).

Results based on the confirmatory sensitivity analyses binomial models (Table 4) demonstrated a decrease in the frequency of consumption for French fries (OR 0.56, 95% CI 0.36, 0.86, *p* = 0.009) and fast food (OR 0.55, 95% CI 0.36, 0.84, *p* = 0.006) among children in the intervention arm. For the control arm, the analysis indicated a decrease in the frequency of consumption of sweets (OR 0.59, 95% CI 0.35, 0.98, *p* = 0.04) and salty snacks (OR 0.59, 95% CI 0.37, 1.00, *p* = 0.05). No decrease in the consumption of sweets or salty snacks was reported by mothers whose child was in the intervention group. 

## 4. Discussion

The present study examined the effect of a culturally-tailored, family-based intervention on the eating behaviors of Hispanic children. After participation in the *Abriendo Caminos* program, mothers in the intervention group reported a decrease in the frequency of their child’s SSBs, French fries, and fast food consumption and an increase in vegetable consumption compared to children in the control group.

SSB consumption is highest among Hispanic children, adolescents, and young adults [39,40]. Data from the National Health and Nutrition Examination Survey revealed that approximately 7% of the total daily calories of Hispanic children come from SSBs [40]. The 2020–2025 Dietary Guidelines for Americans recommend that less than 10% of daily calories come from added sugar [34]. Unfortunately, Hispanic children are almost reaching this percentage with SSBs alone [40]. A meta-analysis that evaluated the effect of interventions targeting SSB intake behaviors among children found that community-based interventions had the greatest impact on reducing consumption [41]. Approximately 82% of our sample population was consuming SSBs at T0 and by T1 this was reduced to 73%. The *Abriendo Caminos* intervention significantly decreased SSBs intake among children in the intervention arm. Changes in SSBs consumption are consistent with findings from other studies that aimed to decrease intake in Hispanic children [10,42,43,44]. A nine-month intervention aimed to improve dietary behaviors in young Hispanic children observed a decrease in SSBs consumption and increased water and milk intake [43]. Similar results have been observed in 7–13-year-old Hispanic children. After a 10 month intervention, children in the experimental arm had modestly lower consumption of SSBs (1.02 daily servings) compared to the control arm (1.39 daily servings) [10].

Fast food and French fries contain high amounts of saturated fat and sodium and are overconsumed in the Hispanic community. Hispanic children’s saturated fat consumption exceeds the 10% recommendation [45]. Therefore, it is imperative to target these eating behaviors, as high consumption of saturated fat and sodium contributes to cardiovascular diseases [46,47,48]. The *Abriendo Caminos* intervention significantly reduced the frequency of fast food and French fry consumption. Our findings corroborate those from other interventions that target Hispanic children. For example, Hispanic children (7–13 years of age) who participated in a four-month intervention led by *promotoras* (community health workers) decreased their fast food consumption [49]. At the end of the intervention, the experimental arm children consumed fast food 1.09 days per week compared to 1.33 days per week for the control arm children [49]. In another study without a control arm, a six-week family-based intervention conducted with a sample of mostly Mexican-American children resulted in a 16.1% reduction in fast food and a 45.2% reduction in French fry consumption [50]. 

A unique finding of the current study was that the frequency of vegetable consumption increased among children in the intervention group compared to control group, as reported by mothers. Likewise, despite having a smaller sample size, the *Abriendo Caminos* pilot program (a single-arm intervention) significantly improved vegetable consumption [9]. However, not all previous nutrition education interventions designed for Hispanic children have observed significant changes in vegetable intake [10,51,52]. A randomized controlled nutrition education intervention for immigrant families (Hispanic, Somali, and Sudanese) did not observe a significant increase in vegetable consumption among adolescents [53]. Arredondo and collaborators found an increase in vegetable variety after a four-month intervention, but they did not assess changes in serving sizes [10]. The food demonstrations and tasting involving vegetables included in the *Abriendo Caminos* may explain the increase in vegetable consumption in our treatment arm. A systematic review and meta-analysis concluded that taste exposure was effective at increasing vegetable intake in short-term interventions [54]. The cultural emphasis of the curriculum on fruits and vegetables commonly consumed in Mexican and Puerto Rican cuisine may also play a role. Nonetheless, we were surprised to find that fruit consumption among children in the intervention group did not change. The reason may be that fruit consumption was already high; at baseline, mothers reported that approximately 60% of children in the intervention arm already consumed fruit two or more times per day. In light of the evidence of high mean HEI-2015 scores for whole fruit (5 out of 5) among Mexican-American children [13], there may be a difficult-to-surmount reason for the lower consumption of fruit among the 40% who did not consume recommended amounts of fruits at baseline.

Another surprising finding was that mothers of children in the control group reported a decrease in the frequency of salty snack consumption post-intervention. During the consent process, families were made aware that they would receive nutrition education materials at the end of the intervention. Therefore, selection bias may play a role for families in the control group who want to make healthy behavior changes. 

### Limitations and Strengths

The present study has several limitations to consider. Similar to other studies that use mothers to report child eating behaviors, our dietary intake metric is subject to social desirability and recall bias. Parents of children who are away from home for more than four hours may not be reliable reporters of their children’s food intake [55]. As such, future studies may want to incorporate an observational design, evaluate the long-term impact(s), and the sustainability of family-based obesity prevention programs with community partnerships. Another limitation is that the measure utilized to assess eating behaviors is not comprehensive, does not report serving sizes and it was not possible to determine changes in the quantities of foods consumed. It also does not evaluate the type and amount of grains or fat intake. Therefore, we cannot assess a child’s diet quality or dietary patterns, as multiple assumptions would need to be made and those inferences will misrepresent the effectiveness of the intervention. Moreover, the survey recall measure did not collect data on water or low-fat milk intake, so it was not possible to determine if a decrease in SSBs leads to an increase in these beverages. However, this screener is a simple measure widely used in comprehensive studies that assess children’s health and can be helpful in settings in which participants have low educational levels, as in this study. Generalizability is also limited as this study predominately included US foreign-born families from Mexico (88%), and only four sites were represented. Thus, it will be essential to develop and evaluate culturally relevant nutrition education and health promotion programming for other Hispanic subgroups.

Despite these limitations, this study has several strengths. This study employed a randomized controlled design to be able to evaluate the effects of the *Abriendo Caminos* intervention. There was a preanalytical plan for the multiple eating behaviors investigated as the outcomes are not independent [25]. In addition, we used the Benjamini–Hochberg procedure as it controls for multiple comparisons. Three of the five negative eating behaviors in the intervention group remained statistically significant: SSBs, French fries, and Fast foods (data not shown). The adjusted *p*-value for vegetable intake changed to 0.06, close to the level of significance set at 0.05. Additionally, results from a multinomial analysis align with the results from the dichotomized models (Supplemental Table S2). The multinomial models concluded that the control group significantly decreased their frequency of consumption of sweets and salty snacks. The intervention group reduced their frequency of consumption for SSBs, French fries, and fast food and increased their frequency of consumption of vegetables. The analyses increased confidence in our results.

The *Abriendo Caminos* curriculum provides evidence-based education on multiple modifiable lifestyle factors that significantly contribute to obesity and incorporates food demonstrations with traditional Hispanic foods where participants can implement knowledge learned in the lessons. This intervention represents a multi-site obesity prevention program that modified eating behaviors related to improvements in Hispanic children’s diet in Illinois, California, Iowa, and Texas. The evidence supplied here suggests that the *Abriendo Caminos* curriculum should be disseminated at a larger scale to help Hispanic families implement healthy dietary behavior changes.

There is a need for more weight management interventions for children already impacted by overweight and obesity. The *Abriendo Caminos* curriculum was developed to prevent, not treat, obesity. In this study, over 50% of the children were affected by overweight or obesity. There is also a need to replicate this study in other rural or urban settings with a broader representation of Hispanic families (e.g., families with heritage from Guatemala, El Salvador, and Nicaragua). Future studies should target Hispanic children earlier in life to prevent the development of obesity, with new methods and metrics. 

Furthermore, a more intensive approach that includes greater integration with the community (e.g., school gardens, farmer’s markets, and community cooking classes), monthly newsletters, and social media channels that reinforce objectives discussed during the intervention may be necessary to help cement healthy eating changes into daily habits and routines. In addition, future work should focus on increasing fathers’ engagement in nutrition programs to help improve the sustainability of dietary changes [56].

## 5. Conclusions

In conclusion, the culturally-tailored, family-based *Abriendo Caminos* program is one of the few randomized controlled studies that demonstrate an increase in the frequency of consumption of nutrient-dense foods and a decrease in consumption of energy-dense foods. The *Abriendo Caminos* curriculum effectively increased the frequency of vegetable consumption and decreased intake of high-fat foods and sodium. The findings from this study can help inform future nutrition education programs that aim to improve dietary behaviors of Hispanic children.

## Figures and Tables

**Table 1:** Abriendo Caminos Weekly Nutrition Lesson Topics.

Week	Topic	Objective	Food Demonstration
1	Portions and Nutrition Facts Label	Understand proper portion sizes for the five major food groups Understand how to read a nutrition facts label Recognize the benefits of drinking water	Cheese quesadilla Water pitcher
2	Fruits and Vegetables	Learn how to incorporate more fruits and vegetables into our plates Understand the importance of fruit and vegetable intake in the context of health	Fruit kabobs Fruits and vegetables seasoned with lemon and chili powder Smoothies with fruits and vegetables
3	Whole Grains and Legumes	Recognize the difference between whole and refined grains Understand how grains benefit our health Identify ways to increase whole-grain intake	Mexicali popcorn Amaranth skulls
4	Salts and Sugars	Identify foods high in sodium Identify healthier, low-sodium alternatives Identify foods high in added sugar Identify sugar alternatives Recognize the consequences of excessive salt and sugar intake	Vegetables seasoned with low-sodium chili powder
5	Fats and Proteins	Identify foods high in fat Identify healthy fats and differentiate them from saturated fats Understand the importance of protein in our diets Understand the differences between plant and animal proteins Identify a variety of lean protein sources	Variety of milk (whole, 2%, 1%, fat-free) Cheese (non-fat, skim, regular)
6	Fiesta	Reflect and apply the information learning in previous weeks by creating and sharing a healthier version of a traditional recipe	Traditional dishes that were modified with healthier ingredients or cooking methods

Adapted from Hannon et al. [25] and Hammons et al. [29].

**Table 2:** Baseline Characteristics of *Abriendo Caminos* Participants.

	Control	Intervention	*p*-Value
Child Characteristics	*n* = 150	*n* = 206	
Age ^1^, years (mean ± SD)	10.2 (3.1)	9.9 (2.7)	0.23
Age Proportion ^2^, % (*n*)			
<12 years	71.33% (107)	80.10% (165)	0.06
≥12 years	28.67% (43)	19.90% (41)	
Girls ^2^, % (*n*)	54.67% (82)	58.25% (120)	0.52
BMI ^3^ percentile categories ^2,4^, % (*n*)			
Normal weight (5th–85th)	40.69% (59)	46.00% (92)	0.57
Overweight (85th–95th)	21.38% (31)	17.50% (35)	
Obese (>95th)	37.93% (55)	36.50% (73)	
Mother Characteristics	*n* = 150	*n* = 206	
Age ^1^, years (mean ± SD)	39.3 (9.1)	39.4 (7.9)	0.91
BMI ^1,3^, kg/m^2^ (mean ± SD)	32.9 (6.8)	32.0 (7.3)	0.28
Weight status ^2^, % (*n*)			
Normal (18.5–24.9 kg/m^2^)	11.41% (17)	12.62% (26)	0.36
Overweight (25.0–29.9 kg/m^2^)	26.17% (39)	33.01% (68)	
Obese (≥30 kg/m^2^)	62.42% (93)	54.37% (112)	
Birthplace ^2^, % (*n*)			
United States	12.84% (19)	10.24% (21)	0.60
Mexico	86.49% (128)	88.29% (181)	
Other	0.68% (1)	1.46% (3)	
Marital Status ^2^, % (*n*)			
Married or Living with Partner	79.33% (119)	79.31% (161)	1.00
Other	20.67% (31)	20.69% (42)	
Education ^2^, % (*n*)			
High School or More	55.15% (75)	53.26% (98)	0.82
Income ^2^, % (*n*)			
$30,000 or more	31.54% (41)	31.05% (59)	1.00
Less than $30,000	68.46% (89)	68.95% (131)	

^1^*p*-values were calculated by independent *t*-test. ^2^*p*-values were calculated by chi-square test. ^3^ BMI: Body mass index. ^4^ Based on age- and sex-specific CDC BMI growth charts [37].

**Table 3:** Generalized Estimating Equation (GEE) Analysis of Child Frequency of Consumption.

	Group		Time		Control ^3^ Group × Time		Intervention ^3^ Group × Time	
Outcomes	OR ^1^ (95% CI ^2^)	*p*-Value	OR (95% CI)	*p*-Value	OR (95% CI)	*p*-Value	OR (95% CI)	*p*-Value
SSBs (*n* = 228)	0.79 (0.42, 1.46)	0.45	0.61 (0.43, 0.85)	0.003	0.67 (0.41, 1.09)	0.11	0.55 (0.35, 0.87)	0.01
100% Fruit Juice (*n* = 225)	0.81 (0.44, 1.49)	0.50	0.97 (0.65, 1.46)	0.89	1.17 (0.62, 2.20)	0.64	0.81 (0.49, 1.34)	0.41
Fruit (*n* = 231)	0.88 (0.58, 1.34)	0.55	1.31 (0.91, 1.90)	0.15	1.43 (0.80, 2.55)	0.23	1.21 (0.77, 1.91)	0.41
French Fries (*n* = 225)	0.84 (0.50, 1.39)	0.49	0.75 (0.55, 1.02)	0.07	1.00 (0.64, 1.57)	1.00	0.56 (0.36, 0.86)	0.009
Vegetables (*n* = 228)	1.15 (0.70, 1.89)	0.59	1.23 (0.81, 1.87)	0.32	0.83 (0.44, 1.57)	0.56	1.84 (1.08, 3.13)	0.03
Fast Foods (*n* = 223)	0.90 (0.55, 1.50)	0.69	0.69 (0.50, 0.95)	0.02	0.86 (0.53, 1.38)	0.53	0.55 (0.36, 0.84)	0.006
Sweets (*n* = 223)	0.93 (0.46, 1.89)	0.83	0.79 (0.49, 1.28)	0.33	0.62 (0.32, 1.22)	0.16	1.00 (0.50, 2.00)	1.00
Salty Snacks (*n* = 224)	0.99 (0.48, 2.05)	0.99	0.54 (0.30, 0.99)	0.05	0.30 (0.11, 0.77)	0.01	1.00 (0.48, 2.07)	1.00

^1^ OR: odds ratio, ^2^ CI: confidence intervals, and ^3^ GEE: generalized estimating equation. GEE binomial models with logit link assessed repeated measures of frequency of consumption at baseline and six weeks post-intervention. Binomial models were adjusted by sex (ref = boys), site (ref = Illinois), child age group (ref = <144 months). Group x time interaction. Boldface type indicates statistical significance *p* < 0.05.

**Table 4:** Sensitivity Analyses with Generalized Estimating Equation (GEE). Binomial Analysis of Child Frequency of Consumption.

	Group		Time		Control ^3^ Group × Time		Intervention ^3^ Group × Time	
Outcomes	OR ^1^ (95% CI ^2^)	*p*-Value	OR (95% CI)	*p*-Value	OR (95% CI)	*p*-Value	OR (95% CI)	*p*-Value
SSBs (*n* = 228)	0.87 (0.55, 1.36)	0.53	0.74 (0.55, 1.00)	0.05	0.74 (0.48, 1.13)	0.16	0.74 (0.49, 1.13)	0.16
100% Fruit Juice (*n* = 225)	0.74 (0.48, 1.16)	0.19	0.93 (0.67, 1.28)	0.65	0.91 (0.58, 1.44)	0.70	0.94 (0.59, 1.49)	0.80
French Fries (*n* = 225)	0.84 (0.50, 1.39)	0.49	0.75 (0.55, 1.02)	0.07	1.00 (0.64, 1.57)	1.00	0.56 (0.36, 0.86)	0.009
Fast Foods (*n* = 223)	0.90 (0.55, 1.50)	0.69	0.69 (0.50, 0.95)	0.02	0.86 (0.53, 1.38)	0.53	0.55 (0.36, 0.84)	0.006
Sweets (*n* = 223)	0.85 (0.56, 1.31)	0.47	0.66 (0.47, 0.92)	0.02	0.59 (0.35, 0.98)	0.04	0.73 (0.47, 1.15)	0.17
Salty Snacks (*n* = 224)	1.27 (0.84, 1.92)	0.25	0.73 (0.51, 1.05)	0.09	0.59 (0.37, 1.00)	0.05	0.91 (0.57, 1.47)	0.71

^1^ OR: odds ratio, ^2^ CI: confidence intervals, and ^3^ GEE: generalized estimating equation. GEE binomial models with logit link assessed repeated measures of frequency of consumption at baseline and six weeks post-intervention. Binomial models were adjusted by sex (ref = boys), site (ref = Illinois), child age group (ref = <144 months). Group x time interaction. Boldface type indicates statistical significance *p* < 0.05.

## Data Availability

The data presented in this study are available upon request from the corresponding author, and five years upon completion of the grant. The data are not publicly available due to concerns regarding privacy.

## Supplementary Materials

The following supporting information can be downloaded at: https://www.mdpi.com/article/10.3390/ijerph19041917/s1, Table S1: Characteristics of Children’s Diet Behaviors; Table S2: Multinomial Analysis of Children’s Eating Behaviors.
