# Supplementary material for: Reducing Obesogenic Eating Behaviors in Hispanic Children through a Family-Based, Culturally-Tailored RCT: Abriendo Caminos
Source: Int J Environ Res Public Health. 2022 Feb 9;19(4):1917. doi: 10.3390/ijerph19041917 (PMC8872523; doi:10.3390/ijerph19041917)
Supplement: Supplementary file 1 [file ijerph-19-01917-s001.zip › ijerph-1509081-supplementary.pdf]

Supplementary Table S1. Characteristics of Children's Diet Behaviors.

	Control				Intervention				*p*-Value [1]
	N	Baseline	n	6-Week Post	n	Baseline	n	6-Week Post	
**SSB**									
0 times per day	16	7.02%	22	9.65%	25	10.96%	39	17.11%	0.84
>0 times per day	76	33.33%	70	30.70%	111	48.68%	97	42.54%	0.83
**Fruit juice**									
0 times per day	18	8.00%	16	7.11%	23	10.22%	27	12.00%	0.66
>0 times per day	73	32.44%	75	33.33%	111	49.33%	107	47.56%	0.83
**Fruit**									
<2 times per day	57	24.68%	49	21.21%	81	35.06%	75	32.47%	0.80
≥2 times per day	38	16.45%	46	19.91%	55	23.81%	61	26.41%	0.78
**French fries**									
0 times per day	30	13.33%	30	13.33%	37	16.44%	54	24.00%	0.32
>0 times per day	65	28.89%	65	28.89%	93	41.33%	76	33.78%	0.42
**Vegetables**									
<2 times per day	70	30.70%	73	32.02%	107	46.93%	92	40.35%	0.38
≥2 times per day	23	10.09%	20	8.77%	28	12.28%	43	18.86%	0.18
**Fast-food**									
0 times per day	28	12.56%	31	13.90%	36	16.14%	53	23.77%	0.50
>0 times per day	64	28.70%	61	27.35%	95	42.60%	78	34.98%	0.56
**Sweets**									
0 times per day	10	4.48%	15	6.73%	17	7.62%	17	7.62%	0.60
>0 times per day	82	36.77%	77	34.53%	114	51.12%	114	51.12%	0.84
**Salty snacks**									
0 times per day	6	2.68%	17	7.59%	14	6.25%	14	6.25%	0.09
>0 times per day	87	38.84%	76	33.93%	117	52.23%	117	52.23%	0.54

[1] *p*-values were calculated chi-square test.

Supplementary Table S2. Multinomial Analysis of Children's Eating Behaviors.

	Group		Time		Control [3] Group × Time		Intervention [3] Group × Time	
**Outcomes**	**OR [1] (95%CI [2])**	***p*-Value**	**OR (95%CI)**	***p*-Value**	**OR (95%CI)**	***p*-Value**	**OR (95%CI)**	***p*-Value**
SSB (n=228)	0.79 (0.52, 1.21)	0.28	**0.67 (0.53, 0.86)**	**0.002**	0.71 (0.50, 1.03)	0.07	**0.63 (0.45, 0.89)**	**0.009**
100% Fruit Juice (n=225)	0.73 (0.48, 1.10)	0.13	0.90 (0.68, 1.19)	0.45	1.04 (0.70, 1.55)	0.85	0.78 (0.53, 1.15)	0.20
Fruit (n=231)	0.93 (0.65, 1.33)	0.68	**1.40 (1.05, 1.89)**	**0.02**	1.51 (0.96, 2.37)	0.07	1.30 (0.89, 1.91)	0.17
French Fries (n=225)	0.91 (0.60, 1.39)	0.66	0.78 (0.60, 1.01)	0.06	0.92 (0.62, 1.36)	0.68	**0.66 (0.46, 0.93)**	**0.02**
Vegetables (n=228)	**1.49 (1.01, 2.18)**	**0.04**	1.10 (0.82, 1.47)	0.52	0.83 (0.53, 1.30)	0.41	**1.46 (1.00, 2.13)**	**0.05**
Fast Foods (n=223)	0.94 (0.62, 1.43)	0.77	**0.76 (0.57, 1.00)**	**0.05**	0.93 (0.60, 1.43)	0.74	**0.62 (0.43, 0.88)**	**0.008**
Sweets (n=223)	0.86 (0.57, 1.30)	0.48	**0.68 (0.51, 0.91)**	**0.01**	**0.56 (0.36, 0.87)**	**0.01**	0.82 (0.55, 1.21)	0.32
Salty Snacks (n=224)	1.24 (0.84, 1.82)	0.28	**0.71 (0.52, 0.98)**	**0.04**	**0.55 (0.34, 0.89)**	**0.02**	0.92 (0.60, 1.40)	0.68

[1] OR: odds ratio, [2] CI: confidence intervals, and [3] GEE: generalized estimating equation. GEE multinomial models with cumulative logit link assessed repeated measures of frequency of consumption at baseline and six weeks post-intervention. Multinomial models were adjusted by sex (ref = boys), site (ref = Illinois), child age group (ref = <144 months). Group x time interaction. Boldface type indicates statistical significance $p < 0.05$.
